# An Electrochemical Nickel–Cobalt (Ni–Co)/Graphene Oxide-Polyvinyl Alcohol (GO-PVA) Sensor for Glucose Detection

**DOI:** 10.3390/s25072050

**Published:** 2025-03-25

**Authors:** Shu-Hui Yeh, Yaw-Jen Chang, Chun-Yi Hsieh

**Affiliations:** Department of Mechanical Engineering, Chung Yuan Christian University, Chung Li District, Taoyuan City 320314, Taiwan

**Keywords:** non-enzymatic glucose sensor, Ni–Co electrodeposition, GO-PVA composite, cyclic voltammetry, linear sweep voltammetry

## Abstract

This paper presents a non-enzymatic sensor for glucose detection in an environment where glucose and insulin coexist. The sensor is based on a three-electrode chip fabricated by etching the copper foil of a printed circuit board. The working electrode is coated with a graphene oxide-polyvinyl alcohol composite film, followed by the electroplating of a nickel–cobalt layer and an additional surface treatment using O_2_ plasma. The experimental results indicate that within a glucose concentration of 2 mM to 10 mM and an insulin concentration of 0.1 mM to 1 mM, the measured current exhibits a linear relationship with the concentration of glucose or insulin, regardless of whether cyclic voltammetry or linear sweep voltammetry is used. However, the detection limit for insulin is 0.01 mM, ensuring that glucose detection remains unaffected by insulin interference. In this sensor, nickel–cobalt serves as a catalyst for glucose and insulin detection, while the graphene oxide-polyvinyl alcohol composite enhances sensing performance.

## 1. Introduction

Glucose is a crucial molecule in the human body, serving as an essential energy source for cells. The concentration of glucose in bodily fluids fluctuates within a range of 4 mM to 7 mM. When the glucose concentration is too low or too high, certain bodily functions become imbalanced, leading to conditions such as diabetes and impaired glucose tolerance. These conditions can result in severe complications in the cardiovascular and renal systems, neurofunctional disorders, and complications such as blindness and coma. Diabetes has become one of the most prevalent diseases globally, characterized by insufficient or inadequate insulin production, making it a chronic metabolic disorder.

According to the World Health Organization (WHO) webpage on diabetes, approximately 200 million people worldwide were diagnosed with diabetes in 1990 [1]. However, in 2022, it was estimated that around 830 million people were affected by the diabetes, causing millions of deaths annually. The prevalence increased from 7% in 1990 to 14% in 2022. WHO predicts that by 2030, diabetes will be one of the leading causes of mortality worldwide. Diabetes has emerged as a significant global public health challenge [2,3].

One of the most distinctive features of diabetes is blood glucose levels. For individuals with diabetes, monitoring and managing blood glucose levels are core methods for reducing long-term complications and improving care for other medical conditions. Therefore, the development of glucose sensors with excellent sensitivity and high selectivity is crucial for glucose detection. Various techniques have been employed for glucose determination, including spectroscopy [4,5,6], electrochemiluminescence [7,8,9], fluorescence [10,11,12], and electrochemistry [13,14,15,16]. Among these, electrochemical detection has gained widespread attention due to its high sensitivity, simplicity, and low cost. Furthermore, the design and fabrication of the sensor itself are critical issues. To enhance sensing sensitivity, various nanostructures, such as nanoparticles, nanorods, and nanoflowers, have been extensively studied [17,18]. Additionally, based on the catalytic mechanism, glucose sensors can be categorized into enzyme sensors and non-enzyme sensors [19,20]. Enzyme sensors operate based on the redox activity of enzymes, such as glucose oxidase (GOx) or glucose dehydrogenase, to measure glucose levels. With their high sensitivity and rapid response, enzymes dominate the commercial glucose sensor market, particularly in electrochemical sensors. However, enzymes are susceptible to denaturation and environmental factors (such as temperature, humidity, and pH), affecting their stability.

In contrast, non-enzyme glucose sensors offer operational simplicity and enhanced stability, overcoming the limitations associated with pH, temperature, and other enzyme-related factors. Various electrode materials, including noble metals [21], transition metals, and their metal oxides [22,23,24], as well as composite materials [25,26], have been used to achieve excellent catalytic activity in non-enzyme glucose sensors. Although noble metals are considered the most important materials for fabricating such sensors, their high cost remains a drawback. As an alternative, transition metals and their compounds have gained attention for non-enzyme glucose detection. Nickel and cobalt, both transition metals, can be electrodeposited as nickel–cobalt (Ni–Co) alloy layers, forming stable deposited films. Compared to noble metals, Ni–Co alloys offer a high-performance yet cost-effective option for electrode fabrication.

Graphene oxide (GO), an oxidized derivative of graphene, contains various hydrophilic oxygenated functional groups, including hydroxyl (-OH), epoxy (-C-O-C-), and carboxyl (-COOH). GO exhibits excellent mechanical properties, a high surface area, and strong chemical stability. It serves as an exceptional filler material, forming strong interactions with polymers to enhance their performance, particularly in biomedical applications. Similarly, polyvinyl alcohol (PVA) is a hydrophilic polymer with a substantial amount of hydroxyl groups along its polymer chains. It is a biocompatible, non-toxic, and bio-degradable polymer with excellent film-forming capabilities. PVA hydrogels exhibit high mechanical strength, an exceptional elastic modulus, and good flexibility. Due to its availability, dielectric strength, and favorable optical properties, PVA has diverse applications in various fields. GO and PVA disperse well in aqueous media, demonstrating high compatibility. Their homogeneous mixture, known as GO-PVA composite, benefits from the high concentration of oxygen-containing groups in GO, which facilitates the formation of additional hydrogen bonds with PVA. This interaction enhances the material’s mechanical properties and thermal conductivity while simultaneously improving its conductivity and sensing performance [27,28,29].

This study investigates the electrodeposition of a Ni–Co coating on a GO-PVA composite film to fabricate a non-enzymatic glucose sensor. Since Ni–Co electroplating is a well-established technique and the preparation of GO-PVA is straightforward, the fabrication process of this sensor is simple and cost-effective. The sensor is designed to detect glucose concentration in environments where glucose and insulin coexist.

## 2. Materials and Methods

### 2.1. Materials

- For sensor fabrication

The chemicals necessary for Ni–Co electrodeposition, such as Ni(NH_2_SO_3_)_2_·4H_2_O, Co(NH_2_SO_3_)_2_·4H_2_O, and others, were sourced from Blue Giant Inc. (New Taipei City, Taiwan). GO was synthesized from graphite powder using a modified Hammer’s method. Subsequently, GO and PVA were introduced into deionized water (DI) and mixed using an ultrasonic oscillator to prepare a GO-PVA aqueous solution with a weight percentage of 5%.

- Bio-materials

The analytes targeted for detection were glucose (C_6_H_12_O_6_, 99.5% extra pure powder, obtained from Sigma-Aldrich (Burlington, MA, USA)) and recombinant human insulin (95% powder, acquired from BioGems International, Inc. (Westlake Village, CA, USA)). In addition, the reagents for the anti-interference study included ascorbic acid (C_6_H_8_O_6_, powder, purchased from Ferak Berlin (Berlin, Germany)), uric acid (C_5_H_4_N_4_O_3_, crystalline, ≥99%, purchased from SIGMA-Aldrich), urea (NH_2_CONH_2_, crystalline, 99%, purchased from SHOWA (Osaka, Japan)), and dopamine (C_8_H_11_O_2_N, powder, ≥ 98%, purchased from SIGMA-Aldrich).

### 2.2. Sensor Fabrication

**Figure 1:**
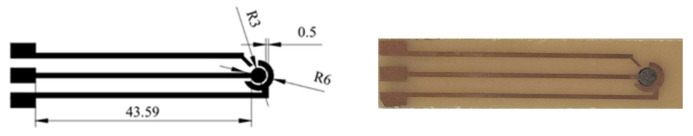
Dimensional design and sensor prototype of the three-electrode chip.

The sensor consists of a three-electrode chip, which includes a working electrode (WE), a counter electrode (CE), and a reference electrode (RE), as shown in Figure 1. The three-electrode chip was fabricated by etching the copper foil of a printed circuit board (PCB) using conventional PCB processing technology. After the fabrication of the three-electrode structure, circuit plating tape was applied, exposing only the working electrode to serve as a masking layer during the electroplating process.

Initially, the chip was treated with O_2_ plasma for 90 s. Subsequently, a 5 μL GO-PVA aqueous solution was drop-cast onto the working electrode, followed by dehydration baking in a 37 °C hot circulator oven for 1 h. The chip was then subjected to an additional O_2_ plasma treatment for 90 s. Following surface modification, a layer of Ni–Co alloy was electroplated onto the GO-PVA.

The electroplating process was conducted in a plating tank with dimensions of 18 cm (width) × 25 cm (length) × 18 cm (height). A pump (100 V/5 W) was installed at the bottom of the tank to generate an upward turbulent flow, ensuring uniform distribution of the plating solution across the metal substrate. Additionally, a filter with a pore size of 1 µm was placed above the pump to remove crystalline solids and impurities.

Three electroplating factors were selected as control variables: electroplating temperature (°C), electroplating current density (A/dm^2^), and electroplating time (min). The Taguchi method was applied using an *L_9_* orthogonal array, as presented in Table 1. Given that insulin is present at a much lower concentration than glucose in the human body, insulin detection was used as the quality characteristic in the Taguchi experimental design and for the subsequent evaluation of sensor performance characteristics. If low insulin concentrations can be detected, the detection of higher glucose concentrations is facilitated. Thus, the Taguchi experiment aimed to determine the optimal electroplating parameters to enhance detection sensitivity. To achieve this, cyclic voltammetry was used to measure insulin at a concentration of 0.5 µM. The ratio of the anodic peak current to its corresponding potential was defined as the outcome variable ψ, following the “Larger-the-Better” design principle. The signal-to-noise (S/N) ratio η is calculated using the following equation:

(1) $$\eta =-10\times log\left(\frac{1}{n}\sum_{i=1}^{n}\frac{1}{\psi_{i}^{2}}\right)$$

where *n* = 3 represents the number of repeated experiments. This method seeks to optimize the electroplating parameters to achieve a higher anodic peak current at a lower potential.

### 2.3. Measurements

The glucose and insulin powders were separately dissolved in phosphate-buffered saline (PBS) containing 0.1 M NaOH to prepare the analyte solutions. Subsequently, these solutions were diluted with PBS to achieve the concentrations required for the experiments.

This study employed two measurement techniques: cyclic voltammetry (CV) and linear sweep voltammetry (LSV). Measurements were performed by depositing 120 µL of the analyte solution onto the sensor’s electrode.

For the CV measurements, cyclic voltammograms were recorded over a potential range from −0.2 V to +1.0 V at a scan rate of 50 mV/s using a plug-and-play potentiostat ECWP100 manufactured by Zensor (Taichung, Taiwan). This potentiostat is compact and equipped with an amplifier circuit that enhances the measured current. In contrast, for the LSV measurements, current-voltage (I-V) curves were obtained over a voltage range of 0 V to 1 V at a scan rate of 40 mV/s using a high-accuracy Keithley 2614B electrometer (Keithley Instruments, Solon, OH, USA).

## 3. Results

### 3.1. Sensor Characteristics

- Characteristics of GO

**Figure 2:**
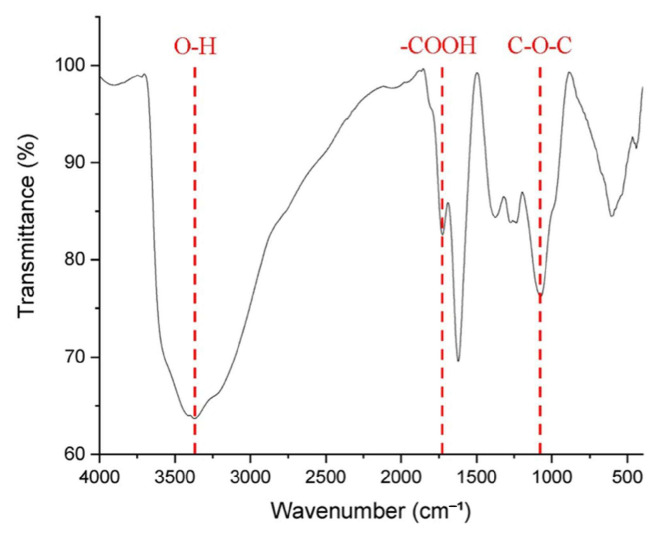
The FTIR spectrum of GO using a JASCO FT/IR-4200. The red dashed lines indicate the absorption bands corresponding to specific functional groups of GO.

The characteristics of GO significantly influence the quality of GO-PVA composites. To confirm the successful preparation of GO, Fourier Transform Infrared Spectroscopy (FTIR) was performed using a JASCO FT/IR-4200 (Tokyo, Japan) within the wavenumber range of 400 cm^−1^ to 4000 cm^−1^, which is typical for organic molecules. The objective was to detect the characteristic functional groups commonly associated with GO. As shown in Figure 2, the FTIR spectrum exhibited a hydroxyl absorption band in the region of 3250–3500 cm^−1^. Since hydroxyl groups readily form hydrogen bonds with water molecules, their quantity directly influences the hydrophilicity of GO. The results revealed a noticeable intensity in the hydroxyl absorption band, indicating that the GO produced in this experiment exhibited sufficient hydrophilicity. This property facilitated its dispersion in deionized water, thereby improving the uniformity of the subsequently prepared GO aqueous solution.

Additionally, absorption bands corresponding to carboxyl and carbonyl groups were observed around 1720 cm^−1^, while an epoxy group absorption band was detected at approximately 1080 cm^−1^. These findings align with those of Kanta et al. [30], confirming the successful oxidation of graphite via the Hummer’s method. Following the preparation of the GO aqueous solution, the oxygen-containing functional groups interacted with the hydroxyl groups of PVA through hydrogen bonding, leading to the formation of a GO-PVA composite film.

**Figure 3:**
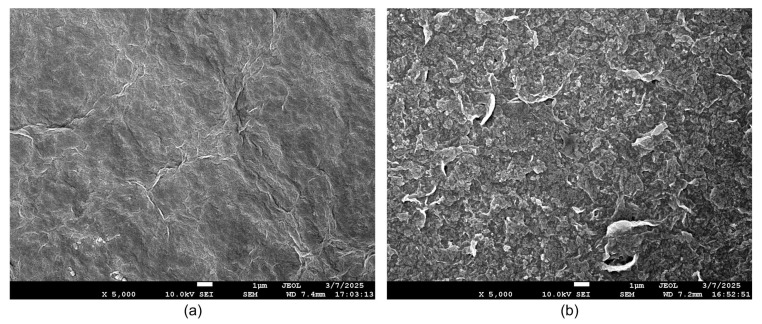
The SEM images using a JEOL JSM-7600F: (**a**) GO. (**b**) GO-PVA.

Furthermore, the SEM images reveal that due to the addition of PVA, the surface morphology of GO-PVA exhibits significant differences compared to that of GO, as shown in Figure 3.

**Figure 4:**
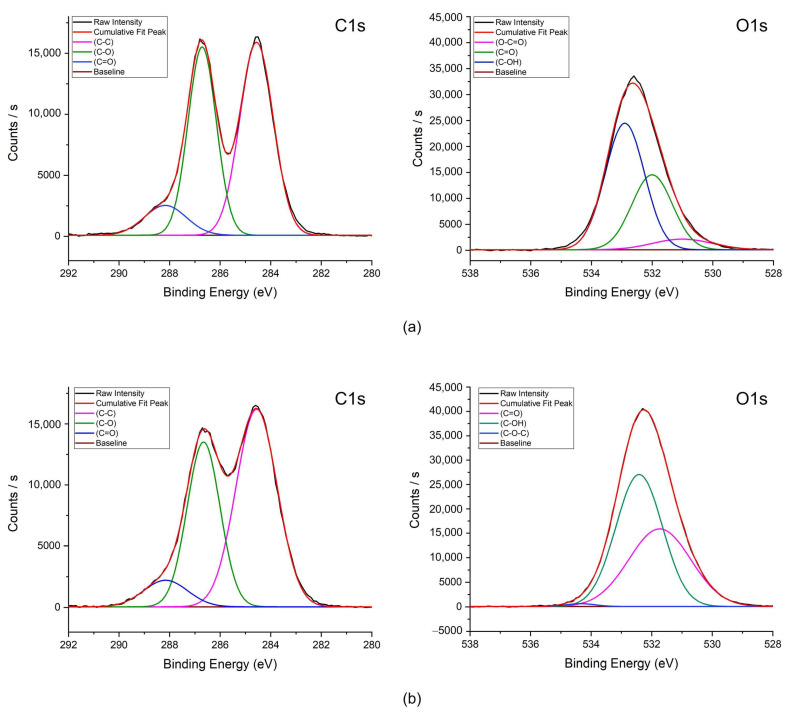
The XPS spectra: (**a**) GO (**b**) GO-PVA.

In addition, Figure 4 shows the XPS spectra of GO and GO-PVA. From the C1s spectrum of GO, the peaks at 284.56, 286.71, and 288.17 eV correspond to C–C (34.40 at.%), C–O (28.28 at.%), and C=O (6.65 at.%), respectively, which represent the primary components of GO. Since PVA mainly contains C–OH groups, the C1s spectrum of GO-PVA shows that the C–O peak (286.66 eV, 23.97 at.%) is influenced by the hydroxyl (-OH) groups of PVA molecules, while the at.% of C=O (288.16 eV, 5.16 at.%) is reduced due to hydrogen bonding interactions between GO and PVA, which partially suppress the formation of C=O bonds.

Similarly, in the O1s spectrum of GO, the peaks at 531.00, 532.00, and 532.90 eV correspond to O–C=O (2.24 at.%), C=O (10.79 at.%), and C–OH (17.64 at.%), respectively. In the case of GO-PVA, the at.% of the C=O (531.73 eV, 15.12 at.%) increases due to the hydrogen bonding effect of PVA, while the intensity of the C–OH peak (532.41 eV, 19.35 at.%) increases, as PVA primarily consists of C–OH alcohol groups. These XPS results confirm the composition of GO-PVA.

- Ni–Co electroplating

**Figure 5:**
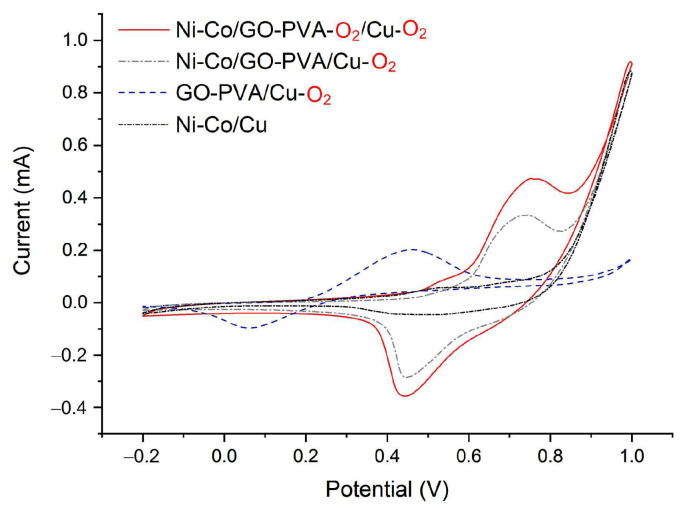
The O_2_ plasma treatment influences the detection of 0.5 mM insulin by enhancing the adhesion of GO-PVA to the electrode and optimizing the Ni–Co electroplating process.

The results of the Taguchi experiment indicated that the optimal electroplating parameters were a temperature of 60 °C, a current density of 5 A/dm^2^, and a duration of 18 min. However, the GO-PVA coating tended to peel off due to the hydrophobic nature of the copper electrode surface. To address this issue, O_2_ plasma treatment was employed to clean the electrode surface and enhance its hydrophilicity before applying the GO-PVA coating. CV measurements using 0.5 µM insulin revealed that O_2_ plasma treatment significantly improved the adhesion of GO-PVA on the copper electrode, facilitating subsequent Ni–Co electroplating. Additionally, the anodic peak current increased to 0.334 mA, as shown in Figure 5, demonstrating that the catalytic effect of Ni–Co on insulin was superior to that of GO-PVA. Notably, when the chip underwent an additional O_2_ plasma treatment before the Ni–Co alloy electroplating, the anodic peak current further increased dramatically to 0.474 mA. These findings clearly indicate that O_2_ plasma treatment enhanced the adhesion of GO-PVA to the copper electrode and improved the Ni–Co electroplating process, thereby increasing its catalytic activity toward insulin.

### 3.2. Detection of Glucose

The normal glucose concentration in the human body ranges from 3.9 to 5.6 mM during fasting and may rise as high as 7.8 mM within two hours postprandially. Therefore, to assess the performance of the Ni–Co/GO-PVA sensor in detecting glucose levels, CV was utilized to measure glucose concentrations ranging from 2 to 10 mM.

Ni–Co has been employed as a catalyst for the detection of glucose, with its catalytic activity primarily relying on the following reactions:

$$Ni+2{OH}^{-}\to {Ni\left(OH\right)}_{2}+2e^{-}$$

$$Co+2{OH}^{-}\to {Co\left(OH\right)}_{2}+2e^{-}$$

$${Ni\left(OH\right)}_{2}+{OH}^{-}\to NiOOH+H_{2}O+e^{-}$$

$${Co\left(OH\right)}_{2}+{OH}^{-}\to CoOOH+H_{2}O+e^{-}$$

$$CoOOH+{OH}^{-}\to CoO_{2}+H_{2}O+e^{-}$$

$$2NiOOH+glucose\to {2Ni\left(OH\right)}_{2}+gluconolactone$$

$$2CoO_{2}+glucose\to 2CoOOH+gluconolactone$$

**Figure 6:**
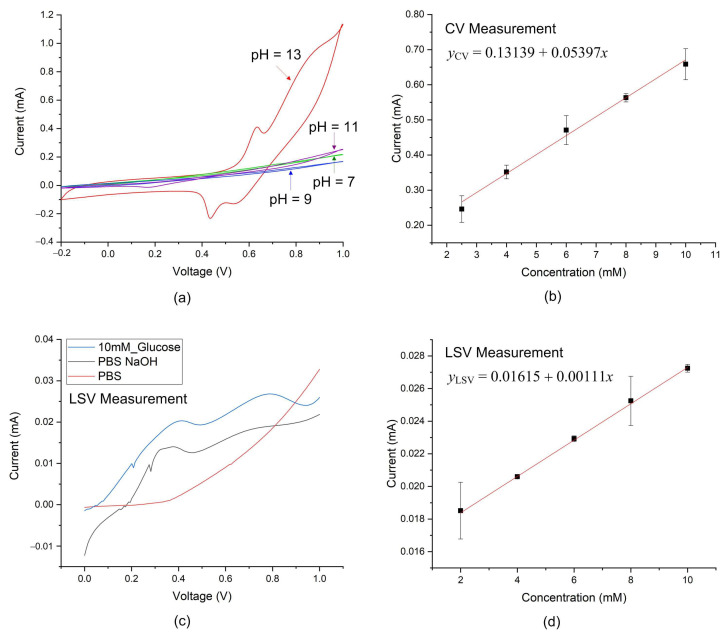
Detection of glucose: (**a**) The effect of pH value on redox reactions. (**b**) Linear relationship between the anodic peak current and glucose concentration obtained from CV measurements. (**c**) Current–voltage curve obtained from LSV measurements. (**d**) Linear relationship between the peak current and glucose concentration obtained from LSV measurements.

To examine the effect of pH value on redox reactions, solutions with different pH values were prepared, and experiments were conducted using CV measurement. As shown in Figure 6a, no redox reaction of glucose was observed when pH ≤ 11. Thus, this study prepared glucose and insulin analyte solutions in PBS (pH = 7.4) containing NaOH (pH = 12.92).

As shown in Figure 6b, the anodic peak current *I_pa_* increases linearly with glucose concentration, indicating that the electrochemical oxidation of glucose was effectively catalyzed by the Ni–Co/GO-PVA sensors. The corresponding linear fitting equation for *I_pa_* can be expressed as:

(2) $$y_{CV}=0.13139+0.05397x,\;\mathrm{with}\;\mathrm{correlation}\;\mathrm{coefficient}\;R^{2}=0.9949$$

where $y_{CV}$ denotes the anodic peak current *I_pa_* (mA) and x is the glucose concentration (mM).

Since the oxidation potential of glucose is approximately 0.65 V, the detection potential for LSV was set in the range of 0 to 1 V. For instance, when measuring 10 mM glucose, the corresponding LSV curve, as shown in Figure 6c, exhibited two distinct oxidation peaks at approximately 0.41 V and 0.75 V. The prominent peak at 0.75 V was attributed to the oxidation of glucose. To investigate the origin of the first prominent peak, the Ni–Co/GO-PVA sensor was employed to test PBS alone and PBS supplemented with NaOH. As shown in Figure 6c, no prominent peaks were observed when testing PBS alone. However, a prominent peak appeared at approximately 0.41 V when testing PBS with NaOH. These results suggest that the first prominent peak observed in Figure 6c originated from the presence of NaOH. Moreover, as shown in Figure 6d, the second oxidation peak in the LSV exhibited a linear relationship with glucose concentration, which can be expressed by the following linear fitting equation:

(3) $$y_{LSV}=0.01615+0.00111x,\;\mathrm{with}\;\mathrm{correlation}\;\mathrm{coefficient}\;R^{2}=0.9993$$

where $y_{LSV}$ denotes the oxidation current *I*_LSV_ (mA) and x is the glucose concentration (mM).

### 3.3. Detection of Glucose with Insulin

**Figure 7:**
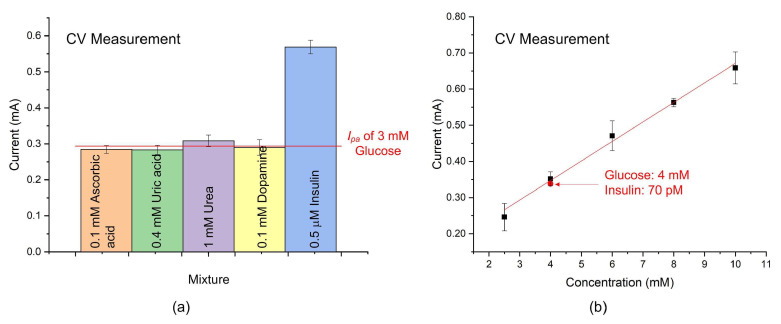
Detection of glucose with insulin: (**a**) Anti-interference study. (**b**) The anodic peak current of a mixture containing 70 pM insulin and 4 mM glucose is approximately equal to that of glucose alone.

In addition to glucose, other substances such as insulin, ascorbic acid, uric acid, urea, and dopamine are present in the blood. The interference of these substances in glucose detection using the Ni–Co/GO-PVA sensor was investigated. Five interference mixtures were prepared by adding 0.1 mM ascorbic acid, 0.4 mM uric acid, 1 mM urea, 0.1 mM dopamine, and 0.5 μM insulin to a 3 mM glucose solution. Each mixture underwent three repeated experiments to observe the electrode’s oxidation reactions and assess its anti-interference capability. As shown in Figure 7a, adding ascorbic acid, uric acid, urea, or dopamine to the glucose solution did not cause any changes in the anodic peak current. The cyclic voltammograms of the glucose solution with added these interfering substances nearly overlap with that of glucose alone, as shown in Figure S1 of the Supplementary Material. Both results indicate that these substances do not interfere with glucose detection when using the Ni–Co/GO-PVA sensor. However, when insulin was introduced, the anodic peak current increased significantly. The detection of glucose is influenced by interference from insulin. This phenomenon can be easily attributed to the sensor’s ability to simultaneously catalyze the oxidation of both glucose and insulin. The following reactions demonstrate that Ni–Co is capable of catalyzing insulin.

$$Ni+2{OH}^{-}\to Ni{\left(OH\right)}_{2}+2e^{-}$$

$$Co+2{OH}^{-}\to Co{\left(OH\right)}_{2}+2e^{-}$$

$$Ni{\left(OH\right)}_{2}+{OH}^{-}\to NiOOH+H_{2}O+e^{-}$$

$$Co{\left(OH\right)}_{2}+{OH}^{-}\to CoOOH+H_{2}O+e^{-}$$

$$NiOOH+insulin\to Ni({OH)}_{2}+oxidizedforminsulin$$

$$CoOOH+insulin\to Co({OH)}_{2}+oxidizedforminsulin$$

Next, the practical applications were considered. The insulin concentration in the blood of a healthy individual on an empty stomach is less than 25 mIU/L (150 pM) [31]. To determine whether the Ni–Co/GO-PVA sensor is affected by insulin interference during glucose detection, this study analyzed a mixture of 70 pM insulin and 4 mM glucose. As shown in Figure 7b, the experimental result indicates that the anodic peak current of the mixed solution in the CV test was approximately the same as that of glucose alone. This is because insulin at the picomolar concentration level generates a signal too weak to be distinguished in the presence of millimolar concentrations of glucose. Furthermore, 0.01 μM insulin can be considered the detection limit of this sensor, as shown in Figure S2 of the Supplementary Material, ensuring that insulin does not interfere with glucose detection by the Ni–Co/GO-PVA sensor.

## 4. Discussion

Although Ni–Co acts as a catalyst for glucose and insulin detection, GO-PVA also plays a crucial role in enhancing the sensor’s performance. As shown in Figure 5, when Ni–Co was directly electroplated onto the copper electrode and tested with 0.5 μM insulin, Ni–Co/Cu exhibited no significant anodic peak current. In contrast, the incorporation of GO-PVA significantly enhanced the sensor’s performance. Furthermore, O_2_ plasma treatment enhanced the adhesion of GO-PVA to the copper electrode, thereby improving Ni–Co electroplating and its catalytic activity.

Since Ni–Co/GO-PVA exhibits superior sensing characteristics compared to Ni–Co /Cu, the measured current and glucose concentration within the range of 2 mM to 10 mM show a linear relationship, regardless of whether a CV or LSV is employed. The potentiostat ECWP100 used for CV measurements is equipped with an amplification circuit, whereas the Keithley 2614B electrometer used for LSV measurements lacks this feature. Consequently, the two methods cannot be directly compared based on the magnitude of the measured current. However, since both glucose and insulin undergo redox reactions, CV measurements are considered more suitable for investigating their redox properties.

Table 2 compares the fabrication processes and glucose detection performance of similar sensors reported in the literature. The proposed sensor is fabricated using a well-established electroplating technique, offering a simple manufacturing process and low production cost.

## 5. Conclusions

Patients with diabetes require continuous monitoring and management of blood glucose levels. This study presents a three-electrode non-enzymatic sensor, where a GO-PVA composite is first coated onto the electrode surface, followed by electroplating with a Ni–Co alloy. The proposed sensor features a simple fabrication process and low cost, eliminating the need for complex nanostructures to enhance sensitivity. The Ni–Co alloy simultaneously catalyzes glucose and insulin, while the GO-PVA composite enhances sensing performance. Notably, the catalytic reaction of insulin does not interfere with glucose detection. This technology demonstrates strong potential for large-scale production.

## Figures and Tables

**Table 1:** Experimental design of the Taguchi analysis.

Factor	Level 1	Level 2	Level 3
Electroplating temperature (°C)	20	40	60
Current density (A/dm^2^)	5	6.5	8
Electroplating time (min)	15	18	21

**Table 2:** Comparative analysis of various sensors developed for glucose.

Sensor Types	Substrate	Fabrication	Linear Range (mM)	Sensibility (μA/mM·cm^2^)	Reference
NiCoO2@CNT	GCE		0.01–1.55	1424.41	[32]
NiCo@*f*-MWCNT		Chemical reduction	Up to 1.6	10,015	[33]
Co/CeO2 nanostructures	GCE	Sol–gel method	0.2–1.0	184	[34]
Ni–Co tungstate	Stainless steel	Successive ionic layer adsorption and reaction	0.05–0.9	18,100	[35]
Ni–Co/GO-PVA	PCB	Electroplating	2–10	763.52	This work

## Data Availability

The data that support the findings of this study are available from the corresponding author upon reasonable request.

## Supplementary Materials

The following supporting information can be downloaded at: https://www.mdpi.com/article/10.3390/s25072050/s1, Figure S1: The cyclic voltammograms of the glucose solution containing interfering substances. Figure S2: Detection of insulin: (a) Segmented linear relationship between the anodic peak current and insulin concentration in the range of 0.01–1 μM, as obtained from CV measurements. (b) Linear relationship between the peak current and insulin concentration in the range of 0.1–1 μM, as obtained from LSV measurements.
