# Supplementary material for: An Electrochemical Nickel–Cobalt (Ni–Co)/Graphene Oxide-Polyvinyl Alcohol (GO-PVA) Sensor for Glucose Detection
Source: Sensors (Basel). 2025 Mar 25;25(7):2050. doi: 10.3390/s25072050 (PMC11991119; doi:10.3390/s25072050)
Supplement: Supplementary file 1 [file sensors-25-02050-s001.zip › sensors-3516749-supplementary.pdf]

An Electrochemical Nickel–Cobalt (Ni–Co)/Graphene Oxide-Polyvinyl Alcohol (GO-PVA) Sensor for Glucose Detection

Anti-Interference Study

Five interference mixtures were prepared by adding 0.1 mM ascorbic acid, 0.4 mM uric acid, 1 mM urea, 0.1 mM dopamine, and 0.5 μM insulin to a 3 mM glucose solution. Except for insulin, the cyclic voltammograms of the glucose solution containing other interfering substances nearly overlaps with that of glucose alone, as shown in Figure S1. This indicates that these substances do not interfere with glucose detection when using the Ni-Co/GO-PVA sensor.

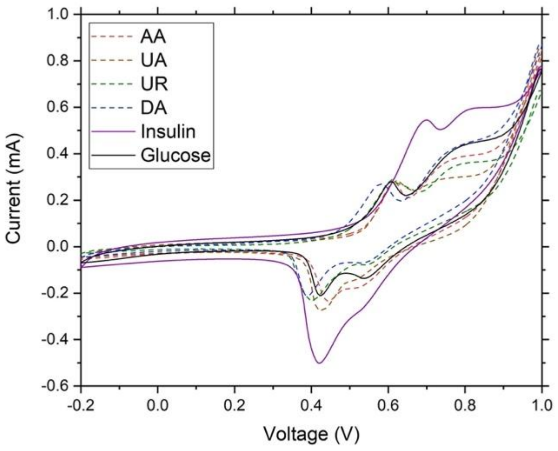

Figure S1. The cyclic voltammograms of the glucose solution containing interfering substances.

Detection of Insulin

In this study, insulin concentrations ranging from 0.01 to 1 μM were measured using both CV and LSV. The experimental results indicate that the anodic peak current in CV exhibits a segmented linear relationship with insulin concentration, as shown in Figure S1(a). Within the concentration range of 0.1 to 1 μM, the corresponding linear regression equation is presented as follows:

$$y_{\mathrm{CV}} = 0.39209 + 0.16814x, \text{ with correlation coefficient } R^2 = 0.9877$$

For the concentration range of 0.01 to 0.1 μM, the linear regression equation is:

$$y_{\mathrm{CV}} = 0.24041 + 1.78141x, \text{ with correlation coefficient } R^2 = 0.9873$$

where y_{CV} denotes the anodic peak current I_{pa} (mA) and x is the insulin concentration (μM). Notably, the sensor's detection capability for insulin concentrations ranging from 0.01 to 0.1 μM decreased significantly. When the concentration dropped below 0.01 μM (5 nM and 7.5 nM in Figure S1(a)), the standard deviation of the anodic peak current in the CV measurements increased. Thus, 0.01 μM can be considered the detection limit of this sensor.

On the other hand, the results of insulin detection using LSV, as shown in Figure S1(b), demonstrate a linear relationship between the oxidation current and insulin concentration within the range of 0.1 to 1 μM. The corresponding linear regression equation is as follows:

$$y_{\mathrm{LSV}} = 0.00188 + 0.00623x, \text{ with correlation coefficient } R^2 = 0.9989$$

where y_{LSV} denotes the oxidation current I_{LSV} (mA) and x is the insulin concentration (μM). However, within the range of 0.01 to 0.1 μM, LSV failed to detect any signal due to the absence of an amplifier circuit in the Keithley 2614B electrometer, resulting in LSV oxidation currents that were too small to measure.

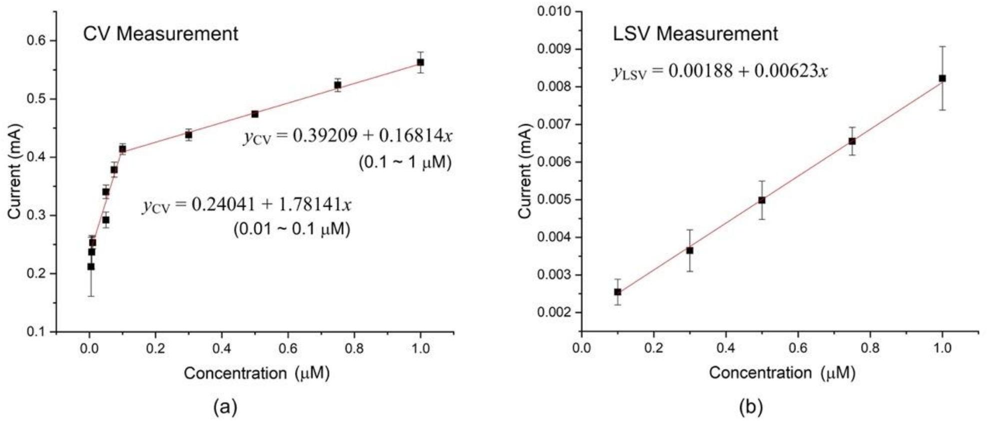

Figure S2. Detection of insulin: (a) Segmented linear relationship between the anodic peak current and insulin concentration in the range of 0.01–1 μM, as obtained from CV measurements. (b) Linear relationship between the peak current and insulin concentration in the range of 0.1–1 μM, as obtained from LSV measurements.
